# High-Complexity *Plasmodium falciparum* Infections, North Central Nigeria, 2015–2018

**DOI:** 10.3201/eid2507.181614

**Published:** 2019-07

**Authors:** Bitrus Yakubu, Ishaya Yohanna Longdet, Horsfall Jen Tony, Dinchi Tyem Davou, Emmanuel Obishakin

**Affiliations:** National Veterinary Research Institute Vom, Jos, Nigeria (B. Yakubu, D.T. Davou, E. Obishakin);; University of Jos, Jos (I.Y. Longdet, H.J. Tony)

**Keywords:** genotypes, genetic, diversity, allele, complexity, infection, *Plasmodium* *falciparum*, nested PCR, malaria, polyclonal, resistance, population displacement, parasites, Nigeria, insurgency, Federal Capital Territory, Abuja, Plateau, Nasarawa, Kaduna, Kogi, *glurp*, *msp1*, *msp2*

## Abstract

The mass migration that occurred during 2009–2013 and after the insurgency in northeastern Nigeria could have increased malaria incidence and *Plasmodium falciparum* genetic diversity in North Central Nigeria. To determine *P. falciparum* sequence diversity in this region, we screened 282 samples collected in regional clinics during 2015–2018 for *Plasmodium* spp. and, with positive samples, determined *P. falciparum* infection complexity and allele diversity using PCR. Of 34 *P. falciparum*–positive samples, 39 *msp1*, 31 *msp2*, and 13 *glurp* alleles were detected, and 88% of infections were polyclonal. We identified trimorphic and dimorphic allele combinations in a high percentage of samples, indicative of a high infection complexity in the study population. High genetic diversity is a catalyst for the evolution of drug-resistant alleles. Improved measures (e.g., better drug quality, diagnostics) are needed to control *P. falciparum* transmission and reduce the potential for the emergence of drug resistance in Nigeria.

Malaria is endemic in Nigeria; its national prevalence among children 6–59 months of age is 27% (*1*). The World Health Organization World Malaria Report 2017 indicated that Nigeria accounted for 27% of the global malaria cases and 30% of the deaths in 2016 (*2*). The insurgency that occurred during 2009–2013 in northeastern Nigeria has been marked as one of the key factors responsible for driving this increased number of malaria cases and deaths; the insurgency disrupted the healthcare system, and up to 2.1 million persons were displaced (*3*–*6*). Added to the insurgency burden were communal conflicts between internally displaced persons (IDPs) and locals, which predominantly occurred in the states of Plateau, Benue, Taraba, Kaduna, and Nasarawa (*3*). Published reports established that 3 patterns of IDP movement transpired in Nigeria: migration to North Central Nigeria (defined as the states of Kwara, Niger, Nasarawa, Plateau, Benue, and Kaduna, and the Federal Capital Territory), economic migration from rural to urban areas, and secondary displacement of host communities because of communal conflicts (*5*).

The population displacement that occurred in North Central Nigeria was likely a key factor affecting the epidemiology of malaria transmission in this region. A high level of new clones of *Plasmodium falciparum* probably would have been introduced into the host communities of towns and cities experiencing influxes of IDPs. Immigration of infected persons into new areas can increase the rate of malaria transmission (*7*) and might subsequently result in infections of higher complexity and pathogens of more extensive genetic diversity in the host communities. In other malaria-endemic areas, population displacement, political unrest, and insurgency have been found to affect malaria epidemiology (*8*–*10*).

Meiotic recombination of *P. falciparum* parasites in *Anopheles* sp. mosquitoes has been proposed as the origin of the generation of novel alleles leading to new *P. falciparum* strains, and this cycle is likely to continue as long as there are vectors, parasites, and human hosts (*11*–*13*). Treatment failures occur more often in patients infected with higher numbers (>3 vs. <3) of *P. falciparum* strains (*14*,*15*). Drug resistance and treatment failures are envisaged to be among the challenges to achieving elimination of malaria in Nigeria (*16*,*17*). Ajayi et al. (*18*) reported 3 cases of artemisinin-based combination treatment failure of *P. falciparum* infection that were later adequately cleared with quinine. However, health practitioners and professionals in Nigeria have determined that the failure to clear parasites and resolve clinical disease after drug treatment is the result of many factors (drug nonpotency, incorrect diagnosis, noncompliance with dosing regimen duration, use of substandard drugs, and drug interactions). These reports spurred us to investigate the complexity of *P. falciparum* infections and *P. falciparum* genetic diversity (allele frequencies) in the North Central region of Nigeria.

Merozoite surface protein 1 (*msp1*), merozoite surface protein 2 (*msp2*), and glutamate-rich protein (*glurp*) gene-based studies of *P. falciparum* genetic diversity and infection complexity have been extensively carried out in other parts of sub-Saharan Africa, but only a few studies have taken place in regions of Nigeria (*19*–*24*). Research is scant on the genetic diversity of *P. falciparum* in North Central Nigeria, and this knowledge is critical for the implementation of successful control measures (*13*). Earlier reports by Jelinek et al. (*25*) and Meyer et al. (*12*) showed that increased genetic diversity of circulating malaria parasites in a population increases the potential for the selection of drug resistance. In our study, we investigated *P. falciparum* genetic diversity and the complexity of *P. falciparum* infections by assessing *msp1*, *msp2*, and *glurp* allele frequencies and genetic diversity in densely populated areas of North Central Nigeria.

## Materials and Methods

### Study Design

**Figure 1:**
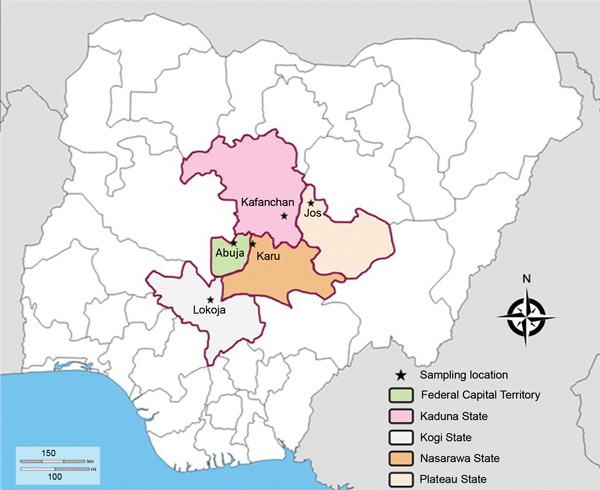
Sampling site locations in study of genetic diversity of *Plasmodium falciparum* spp., North Central Nigeria, 2015–2018. The Jabi region was the sampling site in Abuja.

During August 2015–April 2018, whole blood samples for this study were collected from patients treated at 6 randomly selected healthcare centers in 5 locations of North Central Nigeria: Jos (Plateau State), Karu (Nasarawa State), Kafanchan (Kaduna State), Lokoja (Kogi State), and Abuja (Federal Capital Territory) (Figure 1). We chose these locations because previously published reports indicated that IDP movement occurred in these areas during the peak periods of insurgency, communal conflicts, and economic migration. After collection, whole blood samples were stored at 4°C until we performed PCR analyses at the Department of Biochemistry in the Faculty of Medical Sciences, University of Jos (Jos, Nigeria). Our target was to analyze >50 representative archived whole blood samples from each of the 6 healthcare centers. We received permission to collect and analyze these samples from the National Code for Health Research Ethics Committee.

### PCR Screening for *P. falciparum*

We characterized *P. falciparum* isolates using the nested PCR methods of Snounou et al. (*26*) as modified by Singh et al. (*27*). These modifications included the use of a different genus-specific primer combination (rPLU-1 with rPLU-5 instead of rPLU-5 with rPLU-6) and the use of the nest-1 PCR product as the template for the genus-specific (nest-2) PCR. The primers for these PCRs (Table 1) were designed by using the *Plasmodium* small subunit rRNA genes (*29*) and synthesized by Inqaba Biotec (https://www.inqababiotec.co.za). We performed the nest-1 PCR, then the nest-2 PCR (to identify the samples positive for *Plasmodium* spp.), and then the *P. falciparum* species–specific PCR.

**Table 1:** Primer sequences used for PCRs to screen and genotype samples collected in study of genetic diversity of *Plasmodium falciparum* parasites, North Central Nigeria, 2015–2018*

PCR description	Primer name or type: sequence, 5′→3′	Size, bp	Reference
Nest-1	rPLU1: TCAAAGATTAAGCCATGCAAGTGA	620	(*27*)
	rPLU5: CCTGTTGTTGCCTTAAACTCC		
Nest-2, genus-specific PCR	rPLU3: TTTTTATAAGGATAACTACGGAAAAGCTGT	240	(*27*)
	rPLU4: TACCCGTCATAGCCATGTTAGGCCAATACC		
*Plasmodium falciparum* species–specific PCR	rFAL1: TTAAACTGGTTTGGGAAAACCAAATATATT	205	(*27*)
	rFAL2: ACACAATGAACTCAATCATGACTACCCGTC		
*msp1*, primary reaction	Forward: CTAGAAGCTTTAGAAGATGCAGTATTG	Variable	(*28*)
	Reverse: CTTAAATAGTATTCTAATTCAAGTGGATCA		
*K1*	Forward: AAATGAAGAAGAAATTACTACAAAAGGTGC	Variable	(*28*)
	Reverse: GCTTGCATCAGCTGGAGGGCTTGCACCAGA		
*MAD20*	Forward: AAATGAAGGAACAAGTGGAACAGCTGTTAC	Variable	(*28*)
	Reverse: ATCTGAAGGATTTGTACGTCTTGAATTACC		
*RO33*	Forward: TAAAGGATGGAGCAAATACTCAAGTTGTTG	Variable	(*28*)
	Reverse: CATCTGAAGGATTTGCAGCACCTGGAGATC		
*msp2*, primary reaction	Forward: ATGAAGGTAATTAAAACATTGTCTATTATA	Variable	(*28*)
	Reverse: CTTTGTTACCATCGGTACATTCTT		
*FC27*	Forward: AATACTAAGAGTGTAGGTGCARATGCTCCA	Variable	(*28*)
	Reverse: TTTTATTTGGTGCATTGCCAGAACTTGAAC		
IC/3D7	Forward: AGAAGTATGGCAGAAAGTAAKCCTYCTACT	Variable	(*28*)
	Reverse: GATTGTAATTCGGGGGATTCAGTTTGTTCG		
*glurp*, primary reaction	Forward: TGAATTTGAAGATGTTCACACTGAAC	Variable	(*28*)
	Reverse: GTGGAATTGCTTTTTCTTCAACACTAA		
*glurp*	Forward: TGTTCACACTGAACAATTAGATTTAGATCA	Variable	(*28*)
	Reverse: GTGGAATTGCTTTTTCTTCAACACTAA		

*glurp, glutamate-rich protein; *msp1*, merozoite surface protein 1; *msp2*, merozoite surface protein 2.

The nest-1 reaction mix contained 5 μL of DNA template, 25 μL of One Taq Quick-Load 2× Master Mix (New England BioLabs, https://www.neb.com), 1 μL of each primer (10 μmol/L rPLU1 and rPLU5), and 18 μL nuclease-free water. We used the following cycling program for the nest-1 PCR: initial denaturation at 94°C for 4 min; 35 cycles of 94°C for 30 s, 55°C for 60 s, and 72°C for 60 s; and a final extension at 72°C for 4 min. The nest-2 reaction mix was the same as the nest-1 reaction mix, except that we used 2 μL of the nest-1 PCR product as the template and different primers (rPLU3 and rPLU4). The *P. falciparum* species–specific reaction mix was the same as the nest-2 PCR reaction mix, except that we used different primers (rFAL1 and rFAL2). The cycling programs we used for the nest-2 and *P. falciparum* species–specific PCRs were the same as the one used for the nest-1 PCR, except that we used different annealing temperatures (62°C for the nest-2 PCR and 58°C for the *Plasmodium* species–specific PCR). We performed these amplifications (as well as the genotyping PCR described in the next section) in the GeneAmp 9700 (Applied Biosystems, https://www.thermofisher.com).

### Genotyping by Nested PCR

With *P. falciparum*–positive samples, we performed primary and genotype-specific PCRs for *msp1*, *msp2*, and *glurp* according to the modified method of Snounou and Färnert (*28*). The primary PCRs amplified the block 2 variable region (for *msp1*), block 3 central repeat region (for *msp2*), or the glutamate-rich protein region (for *glurp*). These regions have repetitive segments and motifs that are variable in length among strains; this variability occurs because of the intragenic recombination in *Anopheles* mosquitoes. PCRs targeting these regions amplify sequences of different lengths, which are the basis of clonality in *P. falciparum*. The primary reagent mixture included 5 μL of genomic DNA extract; 25 μL One Taq Quick-Load 2× Master Mix; 1.0 μL of each of the *msp1*, *msp2*, or *glurp* first reaction primers (10 μmol/L) (Table 1); and 14 μL nuclease-free water. We performed amplifications using an initial denaturation at 95°C for 5 min, followed by 25 cycles of denaturation at 94°C for 1 min, annealing at 58°C for 2 min, and extension at 72°C for 2 min and then a final extension at 72°C for 5 min. We used the products of the primary PCRs as the template for the subsequent genotype-specific PCRs, which were used to determine *msp1* (*K1*, *MAD20*, *RO33*), *msp2* (*FC-27*, *IC/3D7*), and *glurp* allele type. The reaction mix and conditions we used for the genotype-specific amplifications were the same as the ones we used for the primary amplification, except that the primers (Table 1), annealing temperature (61°C), and DNA template (2 μL) were different.

### Statistical Analysis

We used GraphPad Prism version 7.03 (https://www.graphpad.com) for allele frequency graph construction. We also used this tool to compare allele frequencies by paired *t* test and analyze the polyclonal distribution of allele families.

## Results

### *P. falciparum* Nested PCR

We obtained 282 archived whole blood samples from the 6 healthcare centers participating in the study. Many of the samples originated from patients who visited the centers for general medical checkups, genotyping, and screening for hepatitis viruses. Various clinical laboratory tests were performed on the collected blood samples as requested by the medical doctors for determining diagnosis or as requested by the patient.

**Figure 2:**
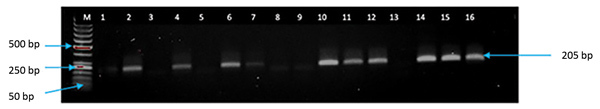
Screening PCR results of persons with *Plasmodium falciparum* parasite infections, North Central Nigeria, 2015–2018. Lane M, 50-bp DNA marker (ThermoFisher Scientific, https://www.thermofisher.com); lanes 1–4, archived blood samples from Nisa Premier Hospital (Jabi, Federal Capital Territory, Nigeria); lanes 5–15, archived blood samples from Kogi Specialist Hospital (Lokoja, Kogi State, Nigeria); lane 16, positive control. Samples positive for *P. falciparum* had a PCR product size of 205 bp.

Of 282 blood samples, 54 were positive for a *Plasmodium* species (240-bp PCR product) by nested PCR and 34 samples were positive for *P. falciparum* (205-bp PCR product; Figure 2). The estimated prevalence of *P. falciparum* among positive samples was 63% (34/54), unusually low compared with the national *P. falciparum* prevalence of ˃95% in 2015 (*1*). However, this finding represents a limited number of samples and cannot truly reflect the national average. The estimated prevalence of 37% for non–*P*. *falciparum* malaria species can probably be attributed to the circulation of >2% of the other *Plasmodium* species that infect humans.

### Allele Diversity and Complexity of Infections

In genotyping PCRs, 32 persons were positive for *msp1*, 29 for *msp2*, and 31 for *glurp*. We found more genetic variation (i.e., more alleles) for the *msp1* gene than the *msp2* or *glurp* genes. We detected a total of 39 *msp1* (16 *K1*, 13 *MAD20*, and 10 *RO33*), 31 *msp2* (16 *FC27* and 15 *IC/3D7*) and 13 *glurp* clones in *P. falciparum*–positive blood samples (Tables 2, 3). A high percentage of the population had evidence of polyclonal infections; >2 different-sized PCR products (i.e., >2 alleles) were present on agarose gel for the *msp1*, *msp2*, or *glurp* genes in 88% of the samples. Similar studies conducted previously in Nigeria (mainly in the southwestern part) during 2004–2014 showed less genetic diversity (Table 3). In those studies, the total number of clones detected were 4 for *K1*, 2 for *MAD20*, and 4 for *RO33* of the *msp1* gene and 9 for *FC27* and 4 for *IC/3D7* of the *msp2* gene. Polyclonal *P. falciparum* infections were more prevalent in our study (multiplicity of infection [MOI], i.e., parasite clones per sample, 2.4) than in the previous reports (MOIs 1.1 and 1.4; Table 3).

**Table 2:** Alleles of genes detected in *Plasmodium falciparum* isolates, North Central Nigeria, 2015–2018*

Allele family	No. positive by PCR	No. alleles	Allele size range, bp	Mean MOI†	% Polyclonal
*msp1*	32				
* K1*	32	16	100–916	2.63	68
* MAD20*	21	13	100–1,200	2.38	73
* RO33*	22	10	100–1,100	2.23	64
*msp2*	29				
* FC27*	17	16	150–1,130	2.52	53
* IC/3D7*	28	15	300–1,200	2.21	68
*glurp*	31	13	200–1,550	1.03	23

*glurp, glutamate-rich protein; MOI, multiplicity of infection; *msp1*, merozoite surface protein 1; *msp2*, merozoite surface protein 2. †Defined as number of parasite clones per sample.

**Table 3:** Distribution of *msp 1*, *msp 2*, and *glurp* clones detected in previous studies conducted in southwestern Nigeria, 2004–2014, and North Central Nigeria, 2015–2018*

Study	Region (state or territory)	No. samples	No. alleles†							Mean MO‡
			*msp1*				*msp2*		*glurp*	
			*K1*	*MAD20*	*R033*		*FC27*	*IC/3D7*		
Happi et al. (*30*)	Southwestern (Ogun)	47	4	2	4		9	4	5	–
Olasehinde et al. (*31*)	Southwestern (Ogun)	100	4	3	1		3	3	–	1.1
Oyebola et al. (*32*)	Southwestern (Lagos)	100	3	2	1		3	3	–	1.4
Bamidele Abiodun et al. (*33*)	Southwestern (Lagos)	78	2	2	1		–	–	–	1.4
This study	North Central (Plateau, Nasarawa, Kogi, Kaduna, FCT)	54	16	13	10		16	15	13	2.4

*FDT, Federal Capital Territory; *glurp*, glutamate-rich protein; MOI, multiplicity of infection; *msp1*, merozoite surface protein 1; *msp2*, merozoite surface protein 2. †Total number alleles found, regardless of size. ‡Defined as number of parasite clones per sample.

**Figure 3:**
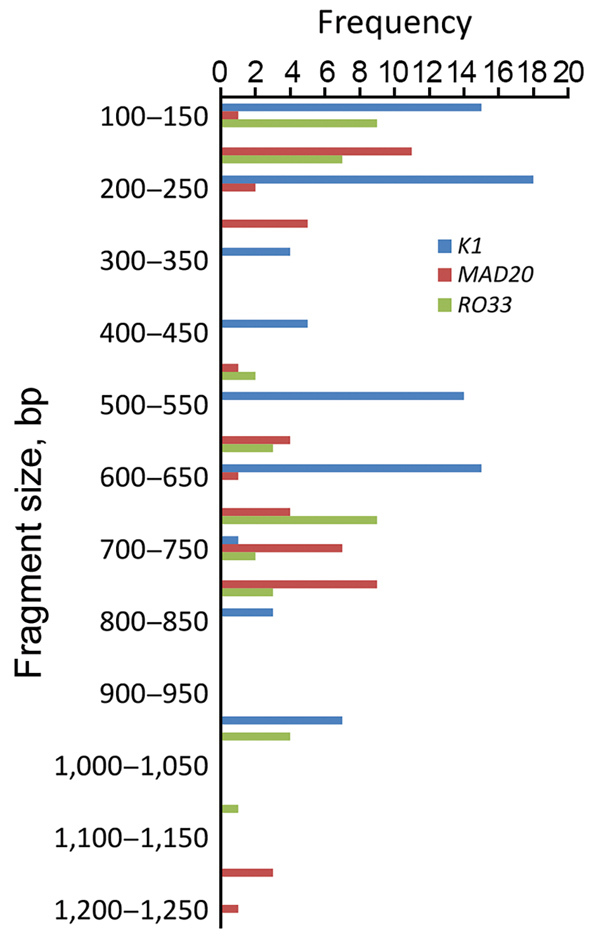
Allele frequency of *msp1* in persons with *Plasmodium falciparum* infection, North Central Nigeria, 2015–2018. The K1 allele of size 200–250 bp was detected at the highest frequency (n = 18). The next highest detected were the *MAD20* allele of fragment size 150–200 bp (n = 11) and the *RO33* alleles of fragment sizes 100–150 bp (n = 9) and 650–700 bp (n = 9).

**Figure 4:**
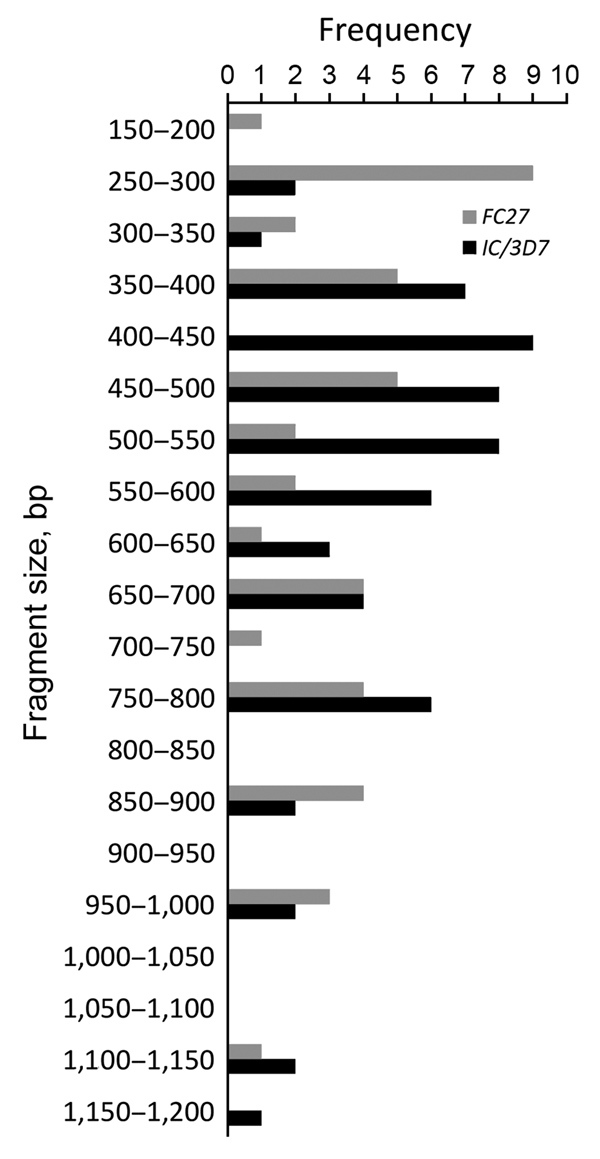
Allele frequency of *msp2* in persons with *Plasmodium falciparum* infection, North Central Nigeria, 2015–2018. The most frequently detected alleles were the *FC27* allele of size 250–300 bp (n = 9) and the *IC/3D7* allele of size 400–450 bp (n = 9).

**Figure 5:**
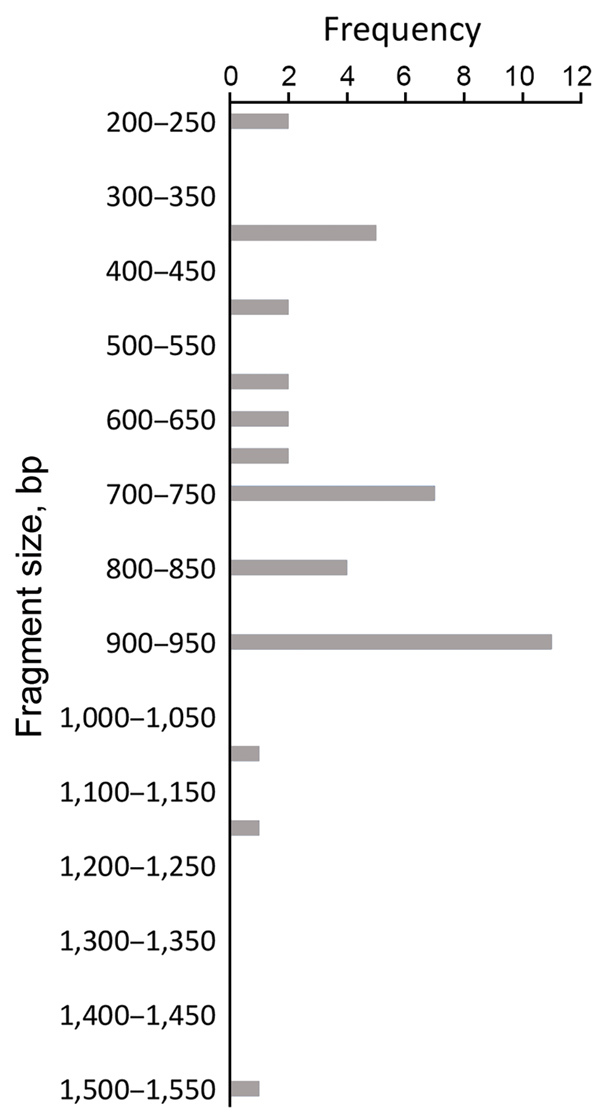
Allele frequency of *glurp* in persons with *Plasmodium falciparum* infection, North Central Nigeria, 2015–2018. The allele with the highest frequency (n = 11) was 900–950 bp in size.

In our study, fragment sizes were 100–1,200 bp for *msp1*, 150–1,200 bp for *msp2*, and 200–1,550 bp for *glurp* (Figures 3,4,5). The highest number of clones seen for a single allele in a single sample was 8 (with 8 clear bands of the *FC27* allele); this sample came from a patient in Kafanchan. A number of samples contained only 1 clone: 8 samples contained a single clone of *K1*, 8 a single clone of *RO33*, 6 a single clone of *MAD20*, 7 a single clone of *FC27*, and 9 a single clone of *IC/3D7*.

The combinations of *msp1* family alleles observed were *K1*, *MAD20*, and *RO33* (63%, 20/32); *K1* and *MAD20* (3%, 1/32); *K1* and *RO33* (6%, 2/32); and *MAD20* and *RO33* (3%, 1/32). Infections were predominantly trimorphic (63%), and fewer were dimorphic (12%); 25% were monomorphic. For the *msp2* allele family, dimorphic infections (*FC27* + *IC/3D7*) occurred in 50% of the population; 35% of the population was positive for only *IC/3D7 *and 15% for only *FC27*. Many patients had infections with high MOIs. The mean MOI per allele family was 2.4 for *msp1* and *msp2* and 1.0 for *glurp* (Table 2). The wide occurrence of trimorphic and dimorphic allele combinations is indicative of high complexity of infection in the study population.

We assessed the frequency of the occurrence of each allele in the population. We categorized clones into molecular weight groups differing by 50 bp for clear discrimination from other clones and elimination of errors that would result from estimating the molecular weight on agarose gels. For the *msp1* allele family, the *K1* allele of size 200−250 bp was detected at the highest frequency (n = 18), followed by the *MAD20* allele of fragment size 150–200 bp (n = 11) and the *RO33* alleles of fragment sizes 100–150 bp (n = 9) and 650–700 bp (n = 9) (Figure 3). For the *msp2* allele family, the most frequently detected alleles were the *FC27* allele of size 250–300 bp (n = 9) and the *IC/3D7* allele of size 400–450 bp (n = 9) (Figure 4). The *glurp* allele with the highest frequency (n = 11) was 900–950 bp in size (Figure 5).

We did not attempt to compare the distribution patterns of the allele families by location because the number of *P. falciparum*–positive samples in some locations was low. However, an analysis of the seasonal patterns of diversity showed that 60% of the infections that occurred during the dry season (November–March) and 50% of those that occurred during the wet season (April–October) were polyclonal. Paired *t* tests showed that the temporal distribution of polyclonal infections and the frequency of clones were statistically significant (p<0.05). During the dry season, the person infected with the highest number of clones (8 clones) was sampled, as well as others infected with 4–5 clones. The finding that polyclonal infections were more common in the dry season was unexpected. However, the increased transmission during this period can be explained by the open gutter and sewage systems present in urban areas of Nigeria; this factor has led to the mosquitoes that potentiate malaria transmission being prevalent year-round.

## Discussion

In this prospective cross-sectional study, we investigated *P. falciparum* genetic diversity and the complexity of *P. falciparum* infection in North Central Nigeria because a high level of genetic diversity and infection complexity was a likely aftermath of the influx of IDPs into the region. We hypothesized that a high level of genetic diversity would be found in the region partly as a result of immigration of *P. falciparum*–infected IDPs into the region. High genetic diversity of malaria parasites circulating in a population could serve as a risk factor for genetic recombination and the generation of novel alleles (*11*–*13*). We found that 63% of the *Plasmodium* spp.–positive samples were positive for *P. falciparum*. Our PCR screening results cannot be used as an estimate of the prevalence of *P. falciparum* in North Central Nigeria, but this finding has revealed that non–*P. falciparum* malaria species are circulating in this region and infecting humans. The finding of 37% positivity for non–*P. falciparum Plasmodium* spp. is high compared with previous reports of low-level prevalence (0.4%–6.3%) in other parts of Nigeria (*30*–*33*). An epidemiologic study of non–*P. falciparum* malaria species circulating in the North Central region would be vital for national malaria control. Treatment failures reported by some authors (*16*,*17*) in Nigeria could be related to incorrect diagnosis and hence inappropriate drug administration. Artemisinin-based combination therapy is recommended for the treatment of *P. falciparum* malaria, but chloroquine plus primaquine is the first-line regimen for *P. vivax* malaria (*34*).

We detected the *msp1*, *msp2*, and *glurp* alleles in most patients positive for *P. falciparum* parasites, and these alleles had high genetic variability. Many samples from infected patients contained multiple alleles, and the genetic variation was more extensive with the *msp1* and *msp2* allele families than with the *glurp* allele. Overall, 88% of the population had evidence of polyclonal infection with >1 of the allelic forms. We discovered 10–16 *msp1* clones and 15–16 *msp2* clones. This finding does not corroborate results of similar previous studies from southwestern Nigeria, in which 1–4 *msp1* clones and 3–9 *msp2* clones were found (Table 3). We detected a total of 39 *msp1*, 31 *msp2*, and 13 *glurp* alleles, which is quite high compared with the reported values in other regions of Nigeria (*30*,*32*) and other countries of West Africa (*21*). Infections with pathogens harboring high numbers of alleles could influence the emergence of resistance (*35*), considering that, in this scenario, multiple alleles are exposed to the same chemotherapeutic agents. The high level of trimorphic, dimorphic, and monomorphic infections in the population is evidence of extensive genetic diversity. Our work revealed a higher frequency of occurrence of polyclonal infections and infections with *P. falciparum* parasites of multiple genotypes, as well as infections of higher MOIs, compared with previous studies (*30*,*33*,*36*).

The question yet to be answered, however, is whether polyclonal infection is a frequent occurrence in Nigeria. The frequent occurrence of this phenomenon in a single geographic area has been determined to be an early indicator for the emergence of alleles associated with drug resistance (*11*,*35*). The high number of polyclonal infections seen in North Central Nigeria could be attributed to a high level of genetic recombination and high evolutionary pressure on the *msp1*, *msp2*, and *glurp* genes in this region, which could partially be attributed to the high level of movement into the North Central region (the economic hub of the nation) for commercial reasons. Drug resistance of *P. falciparum* parasites is a highly complex mechanism involving multiple genes, *pfk13* being a gene responsible (*37*). Work by Oboh et al. (*36*) and Ebenebe et al. (*37*) indicated that in 2018 drug resistance of malaria pathogens to artemisinin-based combination therapy was not an immediate public health threat for southwestern Nigeria. Nonetheless, a nationwide study should be performed to comprehensively evaluate for the presence of *P. falciparum* drug resistance genes with techniques such as next-generation sequencing and genome walking (*38*,*39*). Oboh et al. (*36*) were able to detect synonymous mutations (not validated AA mutations of the *pfk13* gene), such as the nucleotide change from CCG to CCA in the *pfk13* gene in *P. falciparum* from southwestern Nigeria, and called for close monitoring of parasites in Nigeria. A nationwide study should also be conducted to comprehensively determine the genetic diversity of *P. falciparum* and complexity of infections in Nigeria. Determining the genetic diversity of malaria parasites in the North Central region, as well as for the entire nation of Nigeria in general, is needed for the design of a national treatment policy, vaccine development, and immunogenicity studies.

The frequency of polyclonal infections in southwestern Nigeria during 2004–2014 can be described as low and not extensive compared with the level discovered in this study. The period of this study (2015–2018) falls within peak periods of communal conflicts in Nigeria (2009–2017), which resulted in the displacement of persons in the North Central region (*40*,*41*). Our findings suggest a relationship between the high number of polyclonal infections discovered in North Central Nigeria and the movements of IDPs into the region, although a previous study of the *msp2* genotype by Oyedeji et al. (*42*) in Lafia (Nasarawa State) showed a high level of genetic diversity in 2005–2006. A high level of introduction of new *P. falciparum* clones could have occurred in the host communities in the towns and cities that experienced IDP influxes.

A major limitation of this study is the lack of a comparison of the genetic diversity of *Plasmodium* spp. in patients in other regions of the country. However, previous studies conducted in southwestern Nigeria provided a baseline for comparison to determine whether the level of *Plasmodium* spp. genetic diversity discovered in this study was different and thus potentially associated with the movement of IDPs. The correlation would have been more comprehensive if we had also included comparison groups from other regions and specific IDP camps in our investigation.

In summary, this study revealed a high level of *P. falciparum* genetic diversity and infection complexity in North Central Nigeria and suggested that this complexity might be a result of the insurgency and movement of IDPs that occurred in Nigeria during 2009–2017. A deliberate effort is needed to control malaria and eliminate the risk for the evolution of resistance alleles across Nigeria, but particularly in this region of Nigeria, which is the economic hub of the nation and at highest risk for distributing new strains worldwide. The information we generated on *Plasmodium* spp. epidemiology and genetic diversity could serve as a source in a database to be used for policy development on nationwide malaria control and intervention programs for Nigeria. Our work also led to the establishment of a laboratory capable of performing PCR methods to evaluate malaria epidemiology in the region, which could be used for future comprehensive malaria control measures.
